# Better Than Mermaids and Stray Dogs? Subtyping Auditory Verbal Hallucinations and Its Implications for Research and Practice

**DOI:** 10.1093/schbul/sbu018

**Published:** 2014-06-13

**Authors:** Simon McCarthy-Jones, Neil Thomas, Clara Strauss, Guy Dodgson, Nev Jones, Angela Woods, Chris R. Brewin, Mark Hayward, Massoud Stephane, Jack Barton, David Kingdon, Iris E. Sommer

**Affiliations:** ^1^Department of Cognitive Science, ARC Centre of Excellence in Cognition and Its Disorders, Macquarie University, Sydney, Australia;; ^2^Department of Psychology, Durham University, Durham, UK;; ^3^Brain and Psychological Sciences Centre, Swinburne University, Melbourne, Australia;; ^4^Monash Alfred Psychiatry Research Centre, Melbourne, Australia;; ^5^School of Psychology, University of Sussex, Sussex, UK;; ^6^Sussex Partnership NHS Foundation Trust, Sussex, UK;; ^7^Early Intervention in Psychosis, Northumberland, Tyne and Wear NHS FT, Newcastle, UK;; ^8^Department of Psychology, DePaul University, Chicago, IL;; ^9^Centre for Medical Humanities, Durham University, Durham, UK;; ^10^Division of Psychology & Language Sciences, University College London, London, UK;; ^11^Department of Psychiatry, Oregon Health and Science University, Portland, OR;; ^12^Department of Psychiatry, University of Southampton, Southampton, UK;; ^13^Department of Psychiatry, University Medical Center Utrecht, Utrecht, The Netherlands

**Keywords:** AVH, hearing voices, phenomenology, schizophrenia, symptom classification, trauma

## Abstract

The phenomenological diversity of auditory verbal hallucinations (AVH) is not currently accounted for by any model based around a single mechanism. This has led to the proposal that there may be distinct AVH subtypes, which each possess unique (as well as shared) underpinning mechanisms. This could have important implications both for research design and clinical interventions because different subtypes may be responsive to different types of treatment. This article explores how AVH subtypes may be identified at the levels of phenomenology, cognition, neurology, etiology, treatment response, diagnosis, and voice hearer’s own interpretations. Five subtypes are proposed; hypervigilance, autobiographical memory (subdivided into dissociative and nondissociative), inner speech (subdivided into obsessional, own thought, and novel), epileptic and deafferentation. We suggest other facets of AVH, including negative content and form (eg, commands), may be best treated as dimensional constructs that vary across subtypes. After considering the limitations and challenges of AVH subtyping, we highlight future research directions, including the need for a subtype assessment tool.

## Introduction

Auditory verbal hallucinations (AVH: “hearing voices”) occur both transdiagnostically and extradiagnostically.^1–5^ Due to their inherently intriguing nature and ability to cause distress and impairment, the origins of AVH have been sought for millennia.^2^ Yet, their causes remain inexactly understood and clinical interventions incompletely effective. One barrier to progress has been the phenomenological diversity of the experience.^6–10^ No model based on a single mechanism has been able to account for the full phenomenological spectrum of AVH. This is unsurprising given that AVH may be audible or “soundless,” accusing or enthusing, individual or chorus, spoken or sung, recognized acquaintance or anonymous interlocutor, memories of words past or virgin encounters, stilted-repetitive or novel-creative, heard inside the head or perceived in the world, and spoken to or about the person who hears them.^2,8,11–13^ This heterogeneity has led to proposals that subtypes of AVH exist, each underpinned by distinctive (in addition to shared) mechanisms and necessitating tailored interventions.^2,11,14–17^. To operationalize AVH subtypes through identifying differential involvements of specific dimensions of functioning and creating optimally matching interventions is a project that would be consistent with the National Institute of Mental Health’s Research Diagnostic Criteria.^18,19^

Borges wrote of a (likely apocryphal) encyclopedia entry on animals that divided them into (*a*) those that belong to the Emperor, (*b*) embalmed ones, (c) those that are trained, (*d*) suckling pigs, (*e*) mermaids, (*f*) fabulous ones, (*g*) stray dogs, and many more. Such satire reminds us not all subtypes are useful.^20^ Indeed, early AVH subtypes have proven of little practical value to contemporary clinicians and researchers.^2,21,22^ For example, Schneider’s^23^ casting of AVH into those diagnostic of schizophrenia (ie, running commentary and voices conversing) and those not, today fails to predict either diagnosis or need for care.^24^

In order to create psychometrically satisfactory AVH subtypes, reliable clinical description of their essential features is necessary.^25^ Subtypes should hence be able to be clearly operationalized and assessed by structured interview. Validity would be further established by subtypes having distinct neural signatures and being associated with specific patterns of performance on selected cognitive tasks. Additionally, there should be predictive validity,^26^ with a given AVH subtype responding to a specific intervention. Subtypes should also offer a helpful way of making sense of experience, for voice hearers, researchers, and clinicians, although voice hearers’ explanatory frameworks may be incommensurable across other levels of explanation (eg, “spiritual” voices not having different neural correlates to “psychotic” voices). In terms of discriminant validity, subtypes should have some differential associations with external variables such as phenomenological properties, cognitive biases, neural correlates, developmental course, etiological influences, and treatment response.

Equifinality^27^ entails that there may be multiple etiological routes to the same AVH subtype. For example, abusive AVH could have roots in activation of right Broca’s area,^28^ negative self-schema,^29^ or intrusive traumatic memories.^30^ This could then entail different interventions for phenomenologically similar AVH. Conversely, phenomenologically different AVH may have shared psychobiological causes, processes, or mechanisms.^31^ This highlights the need to examine any proposed AVH subtype across multiple levels of explanation and to examine both potential concordances and discordances of subtypes across levels.

## Subtyping by Phenomenology

### Clustering Phenomenological Properties

As with normal voice perception,^32^ AVH perceived as coming from the external world (ie, not within the head) are associated with planum temporale activation.^33^ Other phenomenological properties of AVH may also be approached in this piecemeal manner,^34^ given that the neural correlates of speech perception differ according to whether speech is repetitive or variable,^35^ male or female,^36^ sung or spoken,^37,38^ and familiar or unfamiliar.^39^ Yet, as Dennett^40(p66)^ has noted “You don’t do serious zoology by just strolling through the zoo, noticing this and that, and marvelling at the curiosities.” AVH subtypes have hence been sought through how their phenomenological properties cluster together.

Stephane and colleagues^16^ identified two AVH subtypes in a cluster analysis of 21 phenomenological properties in a sample of 30 people diagnosed with schizophrenia. The first was characterized by having repetitive content, low linguistic complexity (hearing single words), outer space location, clear acoustics, being accompanied by other hallucinations, and attributed to the self. The second had systematized (ie, nonrepetitive) content, high and intermediate linguistic complexity (hearing sentences and conversations), inner space location, multiple voices that were episodic (ie, were not constant), spontaneous (ie, did not have clear triggers), and attributed to another person or source.

More recently, McCarthy-Jones and colleagues,^11^ utilizing a larger sample (*N* = 199) and a different subset of phenomenological properties, found 3 clusters of AVH. The first, “constant commanding and commenting AVH,” represented repetitive, constant commanding and commenting AVH. The second, “replay AVH,” were constituted by being experienced as identical to previously heard words/conversations. The third, “own thought” AVH, did not address the person, spoke in the first person, were similar but not identical to words/conversations that had previously been heard, and were rated as possibly being one’s own voice/thoughts. Although “subtype” suggests mutually exclusive categories, most participants in this study experienced multiple subtypes, making overlapping clusters of co-occurring voice characteristics an alternative conceptualization of these results.

### Other Phenomenological Subtyping

Some approaches have attended to differences in AVH phenomenology associated with differential levels of distress and impairment. For example, voices that issue commands are linked to harm to self and others^41^ and already have specific CBT interventions.^42^ Negative content also offers another salient difference between AVH because this appears more frequent in voice hearers with clinical diagnoses.^43^ Frequency of AVH is a further criterion that relates to levels of associated distress and can also be associated with the severity of AVH.^43^

Voice hearers typically have some form of relationship with their voice, which varies along dimensions associated with normal interpersonal relationships.^44,45^ Some can even engage in a dialog with their voices, speaking to them and getting responses back.^46–48^ Such dynamic AVH^2^ might form a distinct subtype and be harnessed therapeutically to explore meaning associated with voice content.^48^ Yet, here it remains unclear if the ability to converse with a voice is a property of the voice itself (and its associated neurology) or the culture the voice hearer lives within (eg, dialog being encouraged in spiritualist communities).

### Subtypes vs Dimensions

While some phenomenological facets could be viewed as constitutive of an AVH subtype, (eg, being recognized as a memory), others could be viewed as dimensional constructs found to varying extents across different subtypes, eg, commands and negative content. Dimensions such as negative content may be greater in specific AVH subtypes, such as those specifically linked to threat (eg, hypervigilance subtype, below), than in those with no a priori links to specific affective states (eg, epileptic subtype, below). The tension between the instinct to subclassify AVH and the alternative of conceptualizing them along a continuum/dimension or multiple continua/dimensions remains an important issue for further consideration.^49^

## Subtyping by Cognitive Processes

The validity of phenomenologically derived subtypes may increase if they map onto distinct domains of cognition. For example, Replay AVH^11^ may be specifically associated with intentional inhibition and context memory deficits,^50^ and Own Thought AVH^11^ may be seen as being consistent with misattributed inner speech models of AVH that focus on source-monitoring errors for ongoing internal mentation.^51^ A number of proposals have been made for AVH subtyping at the cognitive level.

### Hypervigilance AVH (HV-AH)

HV-AH are proposed to result from an exaggeration of the normally adaptive perceptual bias humans evolved to detect threat.^14^ In this model, an immediate precipitator (eg, stressful life event) triggers an emotionally distressing, aroused state. The person then becomes hypervigilant for threat stimuli, reducing their threshold for detecting threats in the environment and increasing the chance of auditory “false positives” in environmental noise, eg, hearing things that confirm current beliefs around fears of persecution or public exposure of shaming information. This may then lead to the experience of HV-AH, which are phenomenologically characterized as hearing a voice or sounds (eg, laughter or footsteps) with threatening content coming from the external environment. Such threat hypervigilance, as well as ensuing voice hearing, may encourage the development of persecutory ideation, typical of psychosis.

The existence of HV-AH was supported by a recent small-scale cluster analytic study, which found a cluster consisting of voices with an external location and threatening content, occurring while participants’ attention was self-reported as being externally located.^52^ This study also found evidence for another AVH subtype, characterized as occurring in a quiet environment when voice hearers’ attention was internally focused. These AVH could contrastingly be conceived of as having their basis in memories or misattributed inner speech, raising the possibility of subtyping at this level.^7^

### Memory and Inner Speech

Some AVH, due to their mirroring of the developmental purposes of inner speech such as self-regulation of behavior, may have their roots in inner speech.^47,53^ Others may result from the unintentional activation of memories.^50^ An alternative approach begins from cognitive models that conceptualize voices as being rooted in negative cognitions akin to those characteristic of anxiety and depressive disorders.^29,54,55^ If cognitions characteristic of these disorders occurred in the presence of source-monitoring deficits, this could generate discernable AVH subtypes. A lowered capacity for source recognition has already been consistently linked to the tendency to experience hallucinations^56,57^ and is a good candidate for a shared mechanism across many AVH subtypes.

AVH models based upon negative automatic thoughts found in depression,^29,55^ obsessive-like intrusions associated with obsessive-compulsive disorder (OCD)^58^ and trauma-related intrusions associated with Posttraumatic stress disorder (PTSD)^30,59^ have previously been proposed to explain the origins/maintenance of AVH. Clinically, the fit of each of these models appears variable from person to person, suggesting that each may represent a distinct AVH subtype. First, some people experience derogatory voices with ego-syntonic content associated with dysphoria, consistent with a model of voices as verbal representations of negative self-schema^29^ in the same way that self-critical negative automatic thoughts in depression are seen to arise from the activation of negative beliefs about the self.^60^ Second, others experience repetitive intrusive, ego-dystonic voices that are associated with anxiety, disavowed and resisted, potentially giving rise to compulsive behaviors. This is akin to the distressing, repetitive ego-dystonic intrusive thoughts classed as obsessions in OCD. Third, other voices are identical or thematically related to memories of trauma.^59^ Intrusive, vivid memories of trauma play a central role in cognitive models of PTSD where failure to adequately process trauma memories results in repeated, intrusive memories,^61^ and AVH related to trauma could be similarly conceptualized.^30^ Problematically though, the reliving of traumatic experiences in PTSD is usually visual, sometimes olfactory but rarely auditory verbal.^62^ Furthermore, while some memory-based AVH may be the result of dissociative processing during trauma,^46,63^ others may be created through bottom-up activation of neural circuitry associated with verbal memory, not being identified as memories due to context memory deficits.^56^

Basing AVH subtypes in cognitive models of anxiety and depression could lead to subtype-specific interventions. Depressive thoughts and feelings are maintained through rumination,^64^ and compulsive behaviors through metacognitive beliefs about obsessions. Processes relating to depression and anxiety studied in relation to AVH (eg, rumination^65,66^ and metacognition^67^) may inform future psychological interventions for specific AVH subtypes. For example, because trauma-related cognitions in PTSD are maintained by avoidance and safety behaviors,^68^ this offers a psychological strategy for intervention with AVH, as do other techniques from PTSD such as EMDR.^69^

## Subtyping by Neurology

The corollary discharge model of AVH in psychosis is grounded in a specific network of altered connectivity.^70^ Here, alterations to the arcuate fasciculus tract linking Broca’s and Wernicke’s area are proposed to result in self-produced inner speech being experienced as AVH.^71^ It may be hypothesized that this network is associated with AVH that are misattributed forms of inner speech, rather than, eg, intrusive memories. This network of activation appears distinct from the more restricted left posterior language network (temporoparietal and lateral temporal regions) that appears to be associated with AVH in epilepsy, which are also phenomenologically distinct from AVH in psychosis in some aspects.^72^

The phenomenological similarity of continuous (but not episodic) AVH to tinnitus, which is also usually heard continuously, suggests an AVH subtype that at the neural level may be characterized by chronic deafferentation phenomenon of the auditory cortex.^73^ When perceptual areas of the brain are deprived of environmental stimulation, the nervous system tries to compensate for this by increasing sensitivity to minor stimulation. These compensatory mechanisms can create the sensation of true perception, as in Charles Bonnet syndrome. If correct, then focal therapies, eg, transcranial magnetic stimulation (TMS) or transcranial direct current stimulation (tDCS) concentrating on the auditory cortex may be effective for this AVH subtype^74^ because clinical experience suggests that such AVH tend to be poorly responsive to antipsychotic medication.

If there are indeed AVH subtypes identifiable at a neural level, then what do functional magnetic resonance imaging symptom capture studies of AVH^75^ show? Such studies have lent themselves to scanning specific forms of AVH (eg, those that are frequent with a defined onset/offset). However, if a diverse range of AVH subtypes were being imaged here, these studies would be reporting common regions of activation. Future studies should therefore either attempt imaging of specific AVH subtypes or report the AVH phenomenology of participants.

## Subtyping by Causal Antecedents

Kinoshita and colleagues^76^ have proposed subtyping psychosis through distinguishing between causal antecedents, proposing 4 subtypes: stress sensitivity psychosis, traumatic psychosis, anxiety psychosis, and drug-related psychosis. This overarching model may also be applicable to the specific experience of AVH. Etiology-based subtyping is likely to fit well with a transdiagnostic approach. For example, because AVH often arise following traumatic childhood experiences,^21^ it is unsurprising that AVH occur in PTSD,^77^ borderline personality disorder (BPD),^78^ and schizophrenia, which are all diagnoses associated with high rates of childhood trauma.^63,78^ Such trauma-based AVH may form a distinct transdiagnostic AVH subtype. However, PTSD and complex PTSD^79^ may result in AVH with distinctly different phenomenologies, and this requires further investigation.

## Subtyping by Response to Treatment

This section does not focus on differences in response to anticonvulsant and antipsychotic medication as a way to indicate AVH subtypes, as a diagnosis of epilepsy in someone with AVH would be made a priori on the basis of electroencephalography (EEG), rather than trial and error with medication. Antipsychotic resistant and nonresistant AVH may involve different neural mechanisms and/or have different etiological profiles and/or phenomenologies. For example, patients with psychosis who fail to respond to antipsychotic medication have been found not to show increases in striatal dopamine synthesis.^80^ Similarly, levels of striatal dopamine turnover have been found to be normal in people who experienced AVH in the relative absence of delusions.^81^ This underlies the rationale of clinical practice in some countries (eg, the Netherlands) to start antipsychotics only in people with hallucinations *and* delusions. However, it is important to note that the reasons for the failure of pharmacotherapy may be based in individual differences in drug adherence, metabolism, or absorption and that there are often also significant differences in efficacy between and across different antipsychotics. Therefore, response of one type of AVH, but not another, to antipsychotics does not a priori demonstrate the existence of subtypes. Notably, patients with hearing deficits have been reported to show poor response to antipsychotics, suggesting that a deafferentation AVH subtype may not be primarily related to increased dopamine synthesis.^82^ Conversely, the successful response of different AVHs to antipsychotics does not exclude the existence of subtypes, as such drugs may work in different ways for different subtypes, eg, helping individuals with HV-AH due to arousal reduction but aiding other AVH subtypes through salience reduction.

A repetitive AVH subtype may be identifiable at the treatment level, with Stephane and colleagues^15^ reporting 2 patients with repetitive and fixed content (eg, “Do it, hang yourself in the bathroom”) that did not respond to treatment with antipsychotic medications, but which decreased and stopped after treatment with fluvoxamine, a drug known to have antiobsessional effects.

TMS for AVH^83^ has found that people with treatment-responsive AVH are robustly differentiated from nonresponders by having higher pretreatment regional cerebral blood flow in the left STG, with a study^83^ concluding that patients with “higher brain activity in the left STG might constitute a specific clinical subgroup of patients responsive to TMS.” Another study found the greater the coupling between right Broca’s area and Wernicke’s area, the less effective TMS over temporoparietal junction regions was for AVH,^84^ also suggesting the existence of AVH subtypes, potentially with differential involvements of inner speech.

## Subtyping by Diagnosis

AVH phenomenology in BPD and schizophrenia are highly similiar^78,85^ as is the neural activation associated with AVH in schizophrenia and nonpsychiatric populations.^86^ Some diagnoses may hence not be a useful way to subtype AVH.^19^ Yet, the longitudinal course of hallucinations differs between schizophrenia and bipolar disorder,^87^ and AVH in epilepsy are phenomenological and neurally distinct from AVH in schizophrenia^72^ and sometimes respond to anticonvulsants and not antipsychotics,^88^ suggesting that other diagnostic categories are useful for subtyping and guiding treatment. Moreover, some AVH subtypes may be preferentially associated with specific diagnostic entities. For example, HV-AH may be particularly associated with a diagnosis of schizophrenia due to the persecutory ideation that is likely to be associated with threat hypervigilance, memory:dissociative AVH (see below) may be more likely to be found in PTSD, and deafferentation AVH may be especially prominent in those with hearing deficits.

## Subtyping by Voice Hearers’ Own Distinctions

Humans are meaning-making creatures, and people create their own understandings of their voices. Distinctions made by voice hearers may aid AVH subtyping in clinical and research contexts. An interdisciplinary approach to studying narratives of voice hearers (including historical, literary, and ethnographic contexts) could draw out the complexity of how individuals differentiate and classify their experiences, complementing empirical studies of voice hearers’ beliefs about their voices.^89^ A Q-methodology study,^90^ for example, found voice hearers’ explanations could be divided into 6 categories. Three related to perceived origins of the voices (spiritual, psychological, mental illness), and 3 concerned stances toward the voices (resigned pessimist, pragmatic response, passivity to forces). Voice hearer’s own distinction between psychotic and spiritual voices^91^ may prove therapeutically helpful in understanding and helping with the particular forms of distress and impairment arising from AVH.

## Linking Subtypes Across Multiple Levels

The methods through which subtypes are identified exist within hierarchies of validity and usefulness. For example, in a research context subtypes identifiable at the neurological level may be argued to be more valid than those based on self-report measures; in a clinical context, however, some distinctions at the neural level may have little practical relevance for treatment. Consequently, and notwithstanding the potential for fundamental incommensurability between levels of explanation, the greater the concordance across multiple levels, the greater the validity and context-specific utility of a given subtype may be. To date, no studies have been designed to examine concordance across levels, so we here offer a tentative outline as to what such a scheme may look like, amenable to empirical testing.

### Subtype I: Hypervigilance

The HV-AH subtype links the phenomenological and cognitive levels. Although no work has investigated their neurology, they may be associated with activation in neural regions involved in social threat detection (eg, amygdala, orbitofrontal cortex) and threat salience (eg, insula, anterior cingulate cortex). Heightened arousal and focused attention may be detectable as an increase in faster EEG rhythms or as decreased default mode network activity. Clients’ initial coping strategies may be maladaptive (eg, staying awake, sometimes aided by drugs, to protect oneself, and withdrawal from others), exacerbating AVH. A proposed treatment package based on this subtype is in development (Dodgson, in preparation), involving the use of benzodiazepines and specific CBT techniques to reduce threat perception, manage shame, and distract attention. HV-AH could be seen to relate to the anxiety psychosis subtype noted above, and may develop, due to increasing rumination, anxiety and social isolation, into inner speech or deafferentation AVH.

### Subtype II: Autobiographical Memory

Phenomenologically, there is good evidence for a AVH subtype rooted in memories.^11^ These voices, if rooted in highly traumatic events, or in frequent adversity in which the same themes or criticisms were repeated many times with minor variations, may be verbatim replays of what was said. However, given the reconstructive aspect of recall and the tendency to create gist memories,^92,93^ voices may not reflect exactly what was said at the time of the trauma. Based on cognitive models, memory-based AVH may be subdivided into 2 types. Both may evolve over time into more extended, elaborated, novel inner speech–based AVH subtypes.

#### Dissociative.

It has been proposed that a risk factor for PTSD is reduced hippocampal processing of the traumatic event, either because of a preexisting vulnerability or as a response to the intensity of the event. Hippocampal processing would normally integrate information about the event within a spatial and temporal context.^94^ In contrast, decontextualized processing of traumatic events could lead to fragmented, dissociated memories of the trauma with sensory properties, intrusively entering into consciousness as AVH.^21,30,46^ These could be seen to relate to the trauma psychosis subtype previously noted. At a neurological level, as with trauma memories, these may be conceptualized as involving altered functional connectivity between areas such as the amygdala and hippocampus.^95^ These could be treated with trauma-informed psychotherapies and/or EMDR.^69^

#### Nondissociative.

 Memories of speech, which have been processed in a normal (ie, nondissociative) manner, may also intrude into consciousness. These may be experienced as AVH due to deficits in context memory causing them to be experienced as current perceptions rather than memories.^50^

### Subtype III: Inner Speech

#### Obsessional.

Phenomenology suggests the existence of brief, repetitive AVH, which are compelling to act on and often associated with commands. These may be grounded in the obsessional thoughts found in OCD. No neurobiological work has been done on this subtype, yet, the compelling nature of these voices (which appears qualitatively different to typical inner speech) may be based in activation of corticobasal ganglia circuits.^96^ Exposure and response prevention (ERP) may be an intervention particularly suited to this subtype, as may antiobsessional medication.^15^

#### Novel.

The presence of more novel AVH content (eg, running commentaries) may define a subtype, potentially rooted in inner speech.^53^ As with the obsessional subtype, command hallucinations may occur here, suggesting that although commands may necessitate specific forms of treatment, they nonetheless may be treated as a dimensional variable, crossing subtypes. Neurologically, this AVH subtype would fit well with a frontal-temporal corollary discharge model, and interventions involving blocking the phonological loop, reducing rumination, and improving negative self-schema appear likely to be particularly beneficial here, as could tDCS.

#### Own Thought.

These AVH, seen in reports such as “I thought they were really voices but it was really myself thinking to myself”^97(p111)^ have received some empirical support as an AVH subtype.^11^ These may form a way station between normal inner speech and the inner speech:novel AVH subtype. These could be differentiated from other subtypes through the presence of a specific attributional style, requiring therapeutic intervention with specific reattributing techniques.

### Subtype IV: Epileptic

Epileptic AVH appear to be a discrete subtype, being differentiated at the levels of phenomenology, neurology and treatment. Because these should be identified through a clinical diagnosis of epilepsy, we do not discuss them further here.

### Subtype V: Deafferentation

At a phenomenological level such AVH would manifest in continuous (rather than episodic) AVH. The content may frequently be musical but can also be nonmusical. They may be elicited by hearing deficits or social isolation.^73^ Neurologically, they can be conceived of as resulting from deafferentation of the auditory cortex and other language perception areas. The increased resting state activation of the auditory cortex could be best treated with focal therapies such as TMS or tDCS.

## Conceptual Limitations to AVH Subtyping

The majority of AVH subtype research has been performed with people with schizophrenia spectrum diagnoses, potentially obscuring transdiagnostic issues. Research has also been predominantly undertaken in western cultures,^98^ not allowing examination of cross-cultural stability of subtypes. Studying individual symptoms in isolation may also obscure meaningful higher order distinctions. For example, AVH may actually be better studied as part of a class of broader experiences because a continuum can be conceptualized running from clear, externally located AVH,^99^ through internally located AVH with pronounced sensory qualities,^77^ internally located AVH that are experienced as more idea-like than perception-like^11^ “soundless” voices^8^ and into delusions of communication.

We may also ask why AVH have been differentiated from the flux of other experiences associated with psychosis and, to some degree, reified. This distinction is more problematic when we consider AVH whose exact sensory modality is unclear or ambiguous. We should therefore question why and how conceptual boundaries have been “naturalized” over time, and what cultural and/or historical assumptions might continue to support their privileging or centering in clinical and academic discourse.

## Discussion

### Implications for Research

Clearly operationalized AVH subtypes, and assessment methods that reliably and validly identify their presence, need generating. This requires the creation of a bespoke semistructured phenomenological interview designed to identify subtypes because existing tools (eg, Stephane and colleagues^9^ and Carter and colleagues^100^) do not include all questions necessary to characterize subtypes. Questions would need to include enquiries about the location and threat content of the voices, locus of attention during the voice hearing, co-occurring dissociative experiences and persecutory ideation, whether the words are verbatim or thematically related to memories, the repetitiveness of voices, the extent to which the hearer feels compelled to act on the voices’ instructions, presence or absence of dialog, novelty of the content, form of address, and how “voice-like” or “thought-like” the voice is. Cluster analysis could then uncover AVH subtypes. The development of this tool would benefit from voice hearer involvement. Such a schedule should then be employed not only with those diagnosed with schizophrenia spectrum disorders but also with those with PTSD, BPD, or dissociative disorders. Phenomenological subgroups could then inform experimental design^34,101^ and improve signal/noise ratios.^34^ Cognitive tasks (eg, context memory assessment), EEG, and symptom capture imaging studies could then be employed to further examine the unique profiles of these voices, opening the door to tailored psychological and psychopharmacological interventions.

It is also important to consider shared mechanisms across subtypes. Activation of Wernicke’s area has previously been proposed to be a shared mechanism,^34^ and at the cognitive level, inhibitory deficits^102^ may be shared across many AVH subtypes, potentially reflecting altered prefrontal cortex function, as well as source-monitoring deficits.^57^ More work is needed examining commonalities in mechanisms across AVH subtypes.

### Tentative Clinical Implications

Clinical assessment of the AVH subtype(s) a person is complaining of, followed by tailored interventions, appears a promising way forward although it should be acknowledged that some facets of psychological interventions, such as normalization, mindfulness, and compassion-focused therapy^103^ may be beneficial for all AVH subtypes. An understanding of the different cognitive bases for subtypes could facilitate treatment efforts through cognitive remediation. CBT techniques designed to reduce threat perception, manage shame, and distract attention may be useful for HV-AH. Given the association between the Obsessional AVH subtype and the distressing, repetitive intrusive thoughts associated with OCD, and between the memory:dissociative AVH subtype and intrusions associated with PTSD, evidence-based techniques from these other conditions may also be transferable to AVH. For example, for memory:dissociative AVH subtypes, adaptations to trauma-focused CBT or EMDR might help the person to reprocess the trauma memory and reduce AVH distress.^64^ Because ERP has the strongest evidence for effectiveness for OCD, this may be particularly suited to intervening with an obsessional AVH subtype. Clients would be encouraged through ERP to gradually confront the feared actions the voices urge or intimations they make, and resist engaging in compulsive safety behaviors. An example from clinical work (C.S., M.H.) helps illustrate this. Two women reported hearing intrusive voice comments at night telling them their front door was unlocked or their oven was switched on. Believing these comments were warnings they spent hours each night checking their doors and appliances. These could be classed as obsessional AVH. An ERP approach would encourage them to notice these voice comments without checking or with gradually delaying their checking while noting changes in their feelings of anxiety. Habituation may then occur whereby initial feelings of anxiety would lessen over time and the urge to check would gradually reduce.

In addition to psychological interventions, neurostimulation techniques and pharmaceutical agents other than antipsychotics may be useful for specific AVH subtypes, such as benzodiazepines for HV-AH, fluvoxamine for obsessional AVH, and focal therapies for deafferentation AVH.

Case studies offer one way to initially test subtyping hypotheses. After screening potential participants, using a phenomenological subtyping interview of the form proposed above, those found to have only one form of AVH subtype (to avoid the complications of studying multiple subtypes initially) could be invited to participate in a further study. Cognitive assessments could be performed to assess if any predicted cognitive biases are present (eg, biased attention to threat in those with HV-AH; context memory deficits in memory:nondissociative AVH), and, if feasible, symptom capture neuroimaging studies used to determine unique areas of neural activation in different subtypes (eg, frontotemporal networks in inner speech:novel AVH; temporal/temporoparietal regions in epileptic AVH). Tailored interventions, as suggested above, could be trialled within subtypes using a multiple baseline design to explore change over time in purported mechanisms of change and therapy outcomes. Multiple baseline research could lay the groundwork for larger scale trials by identifying potential mechanisms of change and pointing toward which treatments might work most effectively for which subgroups of people hearing voices.

### Conclusions

The identification of AVH subtypes offers the potential to improve our understanding of the causes of AVH and to optimize interventions. Many questions remain though, such as where a dimensional approach may be more appropriate, and how subtypes might evolve from one to another. This area is still in its infancy, and despite the current development of CBT tailored to AVH subtypes, there is much to be understood. Importantly, there is the need for more research into what people who hear voices themselves think are notable distinctions between voices. These ideas, just like Borges’ mermaids and stray dogs, could provide a stimulus to the imagination,^104^ allowing us to better unravel the mystery of AVH.

## Funding

Wellcome Trust Strategic Award (WT098455 to S.M.J. and A.W.); Macquarie University Research Fellowship (to S.M.J.).
